# Meeting of the International Medical Congress at Copenhagen

**Published:** 1884-03

**Authors:** 


### MEETING OF THE INTERNATIONAL MEDICAL CONGRESS, WHICH IS TO BEGIN AT COPENHAGEN.

The time of the meeting of the British Medical Association at Belfast, has been fixed so as not to interfere with the International Medical Congress, which is to begin at Copenhagen on the 10th of August.

A steamer will leave Hull, England, on Aug. 2 and 9 for Copenhagen, and on August 5 a steamer will leave Leith, Scotland, for Copenhagen. Both these places, Hull and Leith, can be reached on any day by leaving Belfast on the previous evening by the cross-channel steamers. Visitors after attending the meeting of the British Medical Association in Belfast, will have ample time to travel to Copenhagen for the Congress.

Communications in reference to the meeting of the British Medical Association at Belfast, to be addressed to the Hon. Local Secretaries John Moore, M.D.; Alex. Dempsey, M.D.; John W. Byers, M.A., M.D.

